# The Role of Cardiac Macrophages in Inflammation and Fibrosis after Myocardial Ischemia-Reperfusion

**DOI:** 10.31083/j.rcm2511419

**Published:** 2024-11-21

**Authors:** Kaiqin Jin, Zijun Ma, Xiaohe Wang, Chen Gong, Jianlong Sheng, Jun Chen, Shichun Shen

**Affiliations:** ^1^Department of Cardiology, Sinopharm Dongfeng General Hospital (Hubei Clinical Research Center of Hypertension), Hubei University of Medicine, 442000 Shiyan, Hubei, China; ^2^Sinopharm Dongfeng General Hospital (Hubei Clinical Research Center of Hypertension), Hubei University of Medicine, 442000 Shiyan, Hubei, China; ^3^Department of Cardiology, The Second Affiliated Hospital of Anhui Medical University, 230001 Hefei, Anhui, China; ^4^Department of Pediatrics, The First Affiliated Hospital of Anhui Medical University, 230001 Hefei, Anhui, China; ^5^Department of Cardiology, The First Affiliated Hospital of USTC, Division of Life Sciences and Medicine, University of Science and Technology of China, 230001 Hefei, Anhui, China

**Keywords:** cardiac macrophages, myocardial ischemia-reperfusion, inflammation, cardiac fibrosis, tissue repair

## Abstract

According to current statistics, the mortality rate of cardiovascular diseases remains high, with coronary artery disease being the primary cause of death. Despite the widespread adoption of percutaneous coronary intervention (PCI) in recent years, which has led to a notable decrease in the mortality rate of myocardial infarction (MI), the pathological cardiac remodeling and heart failure that follow myocardial infarction still pose significant clinical challenges. Myocardial ischemia-reperfusion (MIR) injury represents a complex pathophysiological process, and the involvement of macrophages in this injury has consistently been a subject of significant focus. Following MIR, macrophages infiltrate, engulfing tissue debris and necrotic cells, and secreting pro-inflammatory factors. This initial response is crucial for clearing damaged tissue. Subsequently, the pro-inflammatory macrophages (M1) transition to an anti-inflammatory phenotype (M2), a shift that is essential for myocardial fibrosis and cardiac remodeling. This process is dynamic, complex, and continuous. To enhance understanding of this process, this review elaborates on the classification and functions of macrophages within the heart, covering recent research on signaling pathways involved in myocardial infarction through subsequent MIR injury and fibrosis. The ultimate aim is to reduce MIR injury, foster a conducive environment for cardiac recovery, and improve clinical outcomes for MI patients.

## 1. Introduction

Acute myocardial infarction continues to be a major cause of global mortality, significantly threatening global public health [1]. Acute myocardial infarction generally results from the blockage of coronary arteries that supply blood to the myocardium. In recent decades, mounting evidence has shown that advancements in coronary interventions and thrombolytic treatments have effectively limited infarct size and enhanced clinical outcomes [2]. Particularly, direct percutaneous coronary intervention (PCI) is regarded as the most effective technique to rapidly reestablish myocardial perfusion, minimize ischemic injury, and reduce the exacerbation of infarction [3]. With the widespread adoption of PCI, a new era in the emergency management of myocardial infarction has dawned. Yet, paradoxically, therapeutic reperfusion can cause additional injury through various mechanisms, sometimes further worsening structural and functional injury to the heart [4]. The complications resulting from reperfusion therapy is termed myocardial ischemia-reperfusion (MIR) injury. MIR injury encompass complex interactions between inflammation, oxidative stress, and metabolic factors [5]. Research indicates that MIR injury can result in the expansion of infarct size, with the potential to extend up to half of the total area of infarction [6]. Acute ischemic and hypoxic conditions in the myocardium initiate fibrosis in the necrotic myocardium and cardiac scar formation, which eventually leads to detrimental cardiac remodeling. Moreover, the MIR injury process not only exacerbates myocardial injury but also can lead to widespread cardiac dysfunction, progressing to heart failure.

Inflammation plays a pivotal role in the pathogenesis of cardiovascular diseases, and it is understood that acute inflammation serves as a protective host response to tissue injury or external stimuli [7]. However, as inflammation progresses, in the later stages of MIR, there is significant necrosis of myocardial cells, disruption of cell membranes and organelles, and the release of substantial cellular contents, which further intensifies the inflammatory response [8]. Sustained inflammation (or excessive inflammatory responses) and the inflammation cascade triggered by MIR treatment post-myocardial infarction inevitably lead to continuous injury to the surviving myocardial tissue [9]. A variety of immune cells contribute to the transition from acute inflammation to chronic fibrosis, with macrophages being one of the early recruited cell types during MIR, playing a crucial role in maintaining internal equilibrium and heart development [10]. Recently, the potential of targeting macrophages for treatment, such as altering their phenotypes, has garnered broad interest in the field of cardiovascular disease. This paper explores the heterogeneity of cardiac macrophages, the evolution of macrophage phenotypes in myocardial infarction, the interactions between macrophages and inflammatory responses, the position of macrophages in myocardial fibrosis following MIR, and novel therapeutic approaches to mitigate MIR injury, emphasizing the function of macrophages in post-MIR inflammation and fibrosis and associated emerging treatments.

## 2. Alterations in the Myocardial Tissue Microenvironment Post-MIR

After acute ischemic and hypoxic conditions caused by coronary artery disease, myocardial cells start to die quickly. Once the coronary arteries are re-opened and blood supply to the myocardium is reinstated, MIR injury follows. Early after MIR, myocardial cells face a dual attack from oxidative stress and calcium overload, which compromises cell membrane permeability and disrupts the intracellular environment [11]. At this time, cells release multiple inflammatory factors, attracting immune cells such as macrophages to the myocardial tissue and triggering a sterile inflammatory reaction [12]. Sterile inflammation represents a crucial early event after MIR injury, with the infiltration of inflammatory cells and the release of inflammatory mediators further intensifying the injury and apoptosis of myocardial cells [13]. Subsequently, inflammation activates fibroblasts within the myocardium, prompting these cells to proliferate and produce collagen fibers under the stimulation of inflammatory mediators. With ongoing inflammation and cellular apoptosis, myocardial tissue gradually transitions into the fibrotic remodeling stage [14]. The deposited collagen fibers and other extracellular matrix elements secreted by fibroblasts accumulate within the myocardial interstitium, ultimately impairing cardiac function. This accumulation impacts heart function significantly. Inflammation and fibrosis do not exist in isolation in the aftermath of MIR injury; instead, they influence and exacerbate each other [15]. Inflammation creates the necessary conditions and stimuli for fibrosis, which then aggravates the persistence and deterioration of inflammation. This vicious cycle increasingly worsens cardiac injury, ultimately potentially resulting in severe consequences like heart failure [16]. In the transition process from inflammation to fibrosis, cardiac macrophages serve a regulatory function. The entire process is intricate and precise, with macrophages at different stages displaying various phenotypes, thus transforming the myocardial microenvironment through pro-inflammatory, anti-inflammatory, and fibrotic phases. Hence, understanding the functions of cardiac macrophages and their related mechanisms is particularly crucial.

## 3. The Heterogeneity of Cardiac Macrophages

Macrophages are typically divided into two main categories: classically activated or pro-inflammatory (M1) and alternatively activated or anti-inflammatory (M2) macrophages [17]. Originating from the differentiation of monocytes in bone marrow, they are initially derived from the yolk sac during early development, and become tissue-resident macrophages [18].

Cardiac macrophages arise from two distinct lineages, typically distinguished by differing expressions of C-C chemokine receptor type 2(CCR2). As a receptor for monocyte chemoattractant proteins, CCR2 primarily facilitates the infiltration of monocytes into tissues during inflammation [19]. CCR2- macrophages, mainly located in the heart myocardium, are initially derived from the yolk sac during embryogenesis and are replenished through self-renewal, hence are also referred to as cardiac resident macrophages (CRMs). Another subtype, CCR2+ macrophages, primarily resides in the endocardium, consisting of monocytes from fetal bone marrow post-birth, and are replenished by recruiting circulating monocytes, and are termed infiltrative monocyte-derived macrophages (IMs) [20]. Furthermore, CCR2- macrophages can be further classified into three subgroups based on the expression of major histocompatibility complex II (MHC-II) and lymphocyte antigen 6C (Ly6C), namely MHC-II^high^, MHC-II^low^, and Ly6C+. Thus, cardiac macrophages can broadly be categorized into four subgroups based on surface markers: CCR2-MHC-II^high^, CCR2-MHC-II^low^, CCR2-Ly6C+, and CCR2+ macrophages [21]. Recent research has discovered other surface markers that distinguish between CRMs and IMs, including human leukocyte antigen-DR (HLA-DR),T-cell immunoglobulin and mucin domain containing 4 (TIMD4), lymphatic vessel endothelial hyaluronan receptor 1 (LYVE1). Based on CCR2 and HLA-DR, Geetika Bajpai *et al*. [21] have identified three distinct cell subtypes: CCR2+HLA-DR^high^ and CCR2-HLA-DR^high^ are classified as macrophages, whereas CCR2+HLA-DR^low^ are considered monocytes. According to Dick *et al*. [22], TIMD4+LYVE1+MHC-II^low^ CCR2-macrophages are restored through *in situ* proliferation, TIMD4-LYVE1-MHC-II^high^ CCR2-macrophages are partially replenished by circulating monocytes, and the CCR2+MHC-II^high^ subgroup is completely replenished by monocyte recruitment (Table 1).

**Table 1.** **A brief summary of macrophage classification**.

Subtypes of macrophages	Origin	Lifecycle	Surface markers
CCR2+	Embryonic development	*In situ* proliferation	Ly6C+
			MHC-II^high^
			HLA-DR^high^
CCR2-	Peripheral circulation	Monocyte replenishment	MHC-II^low^
			HLA-DR^high^
			TIMD4
			LYVE1

CCR2, C-C chemokine receptor type 2; Ly6C, lymphocyte antigen 6C; MHC-II, major histocompatibility complex II; HLA-DR, human leukocyte antigen-DR; TIMD4, T-cell immunoglobulin and mucin domain containing 4; LYVE1, lymphatic vessel endothelial hyaluronan receptor 1.

Research on MIR injury has revealed that pro-inflammatory macrophages largely derive from IMs, whereas reparative macrophages are mainly sourced from CRMs [23]. In the infarct area, CRMs constitute approximately 2–5% of the total early-stage cardiac injury macrophages [22]. However, CRMs exhibit a greater capacity to phagocytize fragments and contents of necrotic myocardial cells compared to IMs [20]. Generally, depletion of CRMs triggers adverse cardiac remodeling and left ventricular dysfunction, which ultimately leads to decreased heart function [22]. Additionally, an increase in IMs correlates with the transition of fibroblasts into myofibroblasts, which ultimately results in significant cardiac remodeling [24].

## 4. The Progression of Macrophage Phenotypes Following Myocardial Infarction

Following acute ischemic and hypoxic injury that results in necrosis of myocardial cells, neutrophils and CRMs modulate the infiltration of injured myocardial tissue via numerous circulating Ly6C^high^ monocytes [25]. The population of Ly6C^high^ monocytes quickly surges to a peak, becoming the dominant cell type after myocardial infarction. Swiftly infiltrating Ly6C^high^ monocytes evolve into M1 macrophages, which promote inflammation by engulfing cell debris, breaking down extracellular matrix (ECM), and secreting pro-inflammatory factors (such as tumor necrosis factor alpha (TNF-α), interleukin-1 beta (IL-1β), and interleukin-6 (IL-6)), thus triggering the repair processes after myocardial infarction. Matrix fragments and deceased myocardial cells are removed by macrophages, and the ECM is broken down by secreted matrix metalloproteinases (MMPs) [26], a stage referred to as the inflammatory phase of macrophage clearance of dead cells and debris. The initial inflammatory phase is essential for the repair of heart tissue after MIR injury, yet prolonged inflammation can impede the heart’s recovery [27]. After MIR injury, there is a marked reduction in CRMs due to their depletion. Meanwhile, another type of monocyte, the Ly6C^low^ monocytes, are recruited to the infarcted area of the myocardium and differentiate into M2 macrophages. These cells help mitigate inflammation by secreting anti-inflammatory factors such as IL-10, and they aid in ECM remodeling and angiogenesis [28].

Although this theory is still disputed, transcript analysis of myocardial macrophages after myocardial infarction in mice shows that on day 1, the macrophages have a distinctly pro-inflammatory phenotype, and by day 7, they exhibit a reparative phenotype, consistent with prior theories [29]. Yet, what is different is that both types of macrophages do not exclusively express M1 or M2 markers. For example, macrophages on day 1 also express Arg1, traditionally seen as an M2 marker [30]. Thus, whether M1 and M2 macrophages come from different monocyte groups is still contested. Overall, polarizing macrophages into M1 and M2 may be an oversimplification of a dynamic, complex, and continuous process. Nevertheless, in the early days after myocardial infarction, macrophages with M1-like pro-inflammatory phenotypes predominate, and those with M2 anti-inflammatory phenotypes gradually take precedence in the later stages post-infarction.

In the process of MIR injury, the efferocytosis by macrophages also plays a crucial role. Macrophages maintain myocardial cell homeostasis through the phagocytosis of apoptotic cells or cellular debris [31]. However, the efferocytosis function of macrophages is compromised in MIR, leading to an excessive buildup of necrotic cells, thereby causing secondary necrosis in the heart post-MIR [32]. MerTK is a macrophage receptor that is crucial for clearing dead myocardial cells after MIR. In mouse models of MIR, a deficiency in MerTK results in diminished macrophage clearance capabilities, ultimately leading to an increase in infarct size and reduced cardiac function [33]. Moreover, in the latest study, findings by Xiaohong Wang *et al*. [34] indicate that secreted and transmembrane protein 1a (Sectm1a) serves as a regulator of macrophage efferocytosis in MIR injury. In *Sectm1a* gene knockout mice undergoing MIR, there is noticeable impairment in macrophage efferocytosis, with a concurrent decrease in the ability of macrophages to phagocytize dead cells and a reduction in lysosomal degradation of cell debris. Following this, the apoptotic myocardial cells, which should have been cleared timely, accumulate excessively; this, combined with ongoing severe cardiac inflammation and inappropriate fibrosis, ultimately worsens heart failure. Conversely, therapeutic administration of recombinant Sectm1a protein significantly boosts macrophage efferocytosis, ultimately enhancing cardiac function. Thus, the Sectm1a/Gitr/liver X receptor alpha (LXRα) axis could be a promising approach to augment macrophage efferocytosis for treating MIR injury. We anticipate further studies on macrophage efferocytosis to expand our therapeutic choices.

## 5. The Connection between Macrophages and Myocardial Fibrosis after MIR Injury

Myocardial fibrosis is a critical determinant in the prognosis of myocardial infarction, characterized by the differentiation and proliferation of cardiac fibroblasts and excessive ECM deposition. This process ultimately impairs both the contractile and diastolic functions of the heart, affecting overall cardiac performance [35].

Macrophages play a significant role in the development of myocardial fibrosis following MIR injury. In terms of fibrogenesis, IMs secrete numerous inflammatory (such as TNF-α, IL-1β, and IL-6) and fibrogenic (such as transforming growth factor-beta (TGF-β)) factors [36]. These IMs also facilitate the transformation of cardiac fibroblasts into myofibroblasts, promoting collagen synthesis and subsequent fibrosis. Conversely, CRMs contribute to antifibrosis by upregulating growth factors and ECM components to sustain heart function and prevent the overactivation of cardiac fibroblasts [21]. In the acute phase (1–3 days) post-MIR in the mouse model, the infarct zone exhibits pronounced inflammation, with cytokines IL-1β and IFN-γ initiating an early pro-inflammatory environment following myocardial infarction. Recent studies have shown that inhibiting IL-1β or IFN-γ can decrease cardiac fibrosis and the expression of MMPs [37]. Throughout this process, macrophages strive to repair the heart by releasing various factors, including anti-inflammatory cytokines (such as IL-10), chemokines (such as CC-chemokine ligand 17 (CCL17)), and growth factors (such as TGF-β1) [38]. IL-10, a well-known anti-inflammatory cytokine, significantly reduces the infiltration of inflammatory cells and the expression of pro-inflammatory cytokines in the myocardium. Previous research indicates that myocardial infarction (MI) mice lacking IL-10 exhibit left ventricular dysfunction and increased fibrosis [39]. CCL17, a chemokine that recruits CCR2 macrophages post-cardiac injury, has been found to reduce myocardial fibrosis and enhance left ventricular function in mice lacking this factor [40], suggesting that inhibiting CCL17 could be an effective method to limit myocardial inflammation. The TGF-β1/Smad3 pathway plays a crucial role in cardiac fibrosis remodeling, stimulating fibroblasts to release ECM, which enhances ECM protein synthesis and reduces its breakdown, thus promoting myocardial fibrosis and facilitating myocardial remodeling and repair [41].

In a study by Lavine KJ *et al*. [23], using a diphtheria toxin receptor (DTR)-induced model of myocardial cell injury in mice, it was observed that CCR2+ macrophages exhibited high expressions of TNF-α and IL-1β, while CCR2- macrophages showed notably reduced levels of these cytokines. Additionally, research by Sarah A Dick *et al*. [22] demonstrated that depleting CRMs did not increase acute inflammatory reactions but did lead to deterioration of overall left ventricular contraction, increased myocardial fibrosis, and higher late mortality rates following myocardial infarction.

Activation of the nucleotide-binding oligomerization domain (NOD)-, leucine-rich repeat (LRR)- and pyrin domain-containing protein 3 (NLRP3) inflammasome plays a crucial role in MIR injury. Study suggest that the activation of the NLRP3 inflammasome coincides with the progression of MIR injury by inducing pyroptosis and cardiomyocyte necrosis, and may further exacerbate injury progression through the induction of IL-1β, IL-18, and active caspase-1, ultimately leading to adverse cardiac remodeling [42]. Recent studies have found that activating or inhibiting the NLRP3 inflammasome can either exacerbate or alleviate MIR injury. For example, uric acid can aggravate MIR injury through the reactive oxygen species(ROS)/NLRP3 pathway, the novel NLRP3 inhibitor interferon195 (INF195) can reduce NLRP3-induced pyroptosis in macrophages, and IL-38 can promote the differentiation of M1 macrophages into M2, inhibiting the activation of the NLRP3 inflammasome and thereby mitigating MIR injury [43, 44, 45]. In summary, the NLRP3 inflammasome drives inflammatory cells, including macrophages, which may exacerbate MIR injury. Targeting the NLRP3 inflammasome with specific treatments could potentially alleviate MIR injury to some extent [46].

Legumain (*Lgmn*), a gene uniquely expressed in CRMs, plays a critical role in cardiac inflammation and remodeling. A deficiency in Lgmn results in increased recruitment of CCR2+MHC-II^high^ macrophages and CCR2+MHC-II^low^ monocytes, coupled with a decrease in anti-inflammatory mediators (such as IL-10, TGF-β) and an increase in pro-inflammatory mediators (such as IL-1β, TNF-α, IL-6, IFN-γ). Mice lacking Lgmn exhibited larger areas of infarction 14 days post-MI compared to wild-type mice. Overall, reduced Lgmn levels lead to significantly worse cardiac remodeling after MI, whereas activating Lgmn substantially enhances heart function [47].

Recent research indicates that macrophages can upregulate genes specific to fibroblast ECM structures (such as Postn), thereby gaining the capability to adopt a fibroblast-like phenotype [48]. Moreover, macrophages might directly induce fibrosis by secreting ECM proteins. Thus, stimulating macrophages to produce ECM could serve as a potential therapeutic target for myocardial fibrosis, although further studies are necessary. Previous research targeting pro-fibrotic M2 macrophages has demonstrated that, during the onset of fibrosis in MIR mice, depleting M2 macrophages not only lessened myocardial fibrosis injury but also provided some cardiac protection [49]. However, depleting M2 macrophages during the inflammation resolution and tissue repair phases resulted in deteriorated cardiac function and exacerbated inflammation [50, 51].

Shichun Shen *et al*. [52] discovered in MIR mice that granulocyte-macrophage colony-stimulating factor (GMCSF) activates the CCL2/CCR2 signaling pathway, which enhances NLRP3/caspase-1/IL-1β-mediated inflammatory injury and promotes M1 macrophage polarization. Additionally, CCL2 mediates the transformation of CCR2+ macrophages into the M2 phenotype, leading to the release of TGF-β, which promotes myocardial fibrosis. Inhibition of CCR2 (through gene knockout in mice or pharmacological inhibition) can reduce inflammatory mediators and suppress NLRP3/caspase-1/IL-1β signaling, thereby mitigating MIR injury and presenting a novel therapeutic approach for myocardial fibrosis after MRI injury.

Since myocardial infarction causes irreversible damage to adult cardiac cells, maximizing myocardial regeneration after MIR injury becomes particularly important. Konfino* et al*. [53] observed that macrophages transiently accumulate at the site of myocardial resection and subsequent regeneration in neonatal mice, which to some extent demonstrates a correlation between macrophages and cardiac regeneration. Wang *et al*. [54] found that in a neonatal mouse model of myocardial infarction, a stronger fibrotic response was observed on the seventh day after birth compared to the first day, particularly marked by a significant proliferation of fibroblasts. Aurora *et al*. [55] found that on the first day after birth, macrophages are abundantly and evenly distributed throughout the heart following myocardial infarction, whereas by the fourteenth day, the number of macrophages is reduced and confined to the infarct area. Lavine *et al*. [23] discovered that CRMs play a central role in mediating cardiac regeneration in neonatal mice. Therefore, modulating the function of specific macrophage subgroups and fibroblasts following myocardial infarction may become an effective therapeutic approach to promote cardiac regeneration in the future, but further research is still needed to substantiate this.

**Fig. 1.**
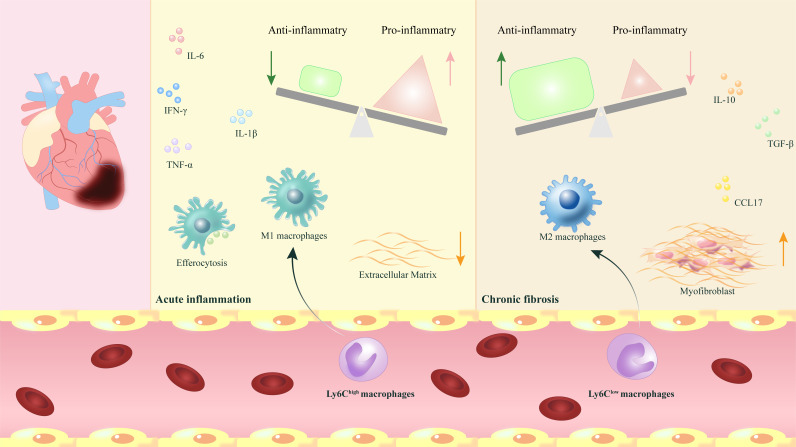
**The phenotypic transformation and release of myocardial macrophages play regulatory and reparative roles following myocardial ischemia-reperfusion**. After myocardial ischemia-reperfusion (MIR), the phenotypic transformation of myocardial macrophages and the release of various pro-inflammatory and anti-inflammatory factors play a crucial role in dynamically regulating the transition from acute inflammation to chronic fibrosis, which is vital for the repair process following MIR. IL-6, interleukin-6; IL-10, interleukin-10; IL-1β, interleukin-1 beta; IFN-γ, interferon-gamma; TNF-α, tumor necrosis factor; TGF-β, transforming growth factor-β; CCL17, CC-chemokine ligand 17; Ly6C, lymphocyte antigen 6C.

In conclusion, the process of myocardial fibrosis is sophisticated and delicate, involving various macrophage subgroups that participate in and regulate each other. Current studies suggest that the role of macrophages in cardiac function regulation might be a prospective research focus, yet the mechanisms involved are highly complex and necessitate further investigation. Thus, a thorough comprehension of the types and functions of different macrophage subgroups offers opportunities to enhance future clinical MIR treatments (Fig. 1).

## 6. New Therapeutic Developments in Macrophages for Reducing Inflammation and Fibrosis Post-MIR

Even though interventional therapies and drug interventions can slow down cardiac remodeling post-myocardial infarction, the excessive inflammation and myocardial fibrosis following MIR inevitably lead to adverse cardiac remodeling. Increasing research has demonstrated that macrophages are crucial for balancing inflammation and fibrosis following MIR injury, making targeted therapies at macrophages a focal point of interest. As precise targeting strategies for macrophages evolve, there is potential to significantly reduce the cardiac injury caused by MIR.

### 6.1 MicroRNA Therapy Based on Exosome Delivery

Mesenchymal stem cells (MSCs) have garnered widespread attention due to their high replicative potential, low immunogenicity, and capacity for multilineage differentiation [56]. Studies have shown that MSC-derived exosomes (MSC-exos) protect against inflammation and enhance cardiac function after MIR injury by suppressing inflammatory responses [57, 58]. MSC-exos contain multiple components, and certain microRNAs within MSC-exos have been identified as key regulators of immune responses. Zhao *et al*. [59] found that injecting MSC-exos into the myocardium of mice following MIR injury regulated and inhibited toll-like receptor 4 (TLR4) via miR-182, facilitating the transition from M1 to M2 macrophages, thereby reducing infarct size and alleviating cardiac inflammation. miR-125a-5p, highly concentrated in MSC-exos, enhances M2 macrophage polarization and decreases fibroblast proliferation and activation, thereby improving inflammation and reducing apoptosis in myocardial cells [60]. Furthermore, microRNAs such as miR-101a [61], miR-181b [62], and miR-155 [63] have also demonstrated capabilities in regulating macrophage polarization, controlling inflammatory responses, and promoting cardiac remodeling following MIR injury. Overall, exosome-based microRNA therapy is an emerging treatment with vast potential for modulating inflammatory responses and enhancing cardiac remodeling post-MIR injury. Nevertheless, significant challenges remain, such as the short half-lives of exosomes, dosing complexities, the absence of standardized isolation and purification methods that ensure bioactivity, and the risk of capture by the mononuclear phagocyte system following intravenous administration [64, 65]. Thus, extensive research is necessary to thoroughly assess exosome-delivered microRNA therapies and optimize outcomes.

### 6.2 Therapy Based on Nanomaterial-Mediated Drug Delivery 

Nanomedicine has emerged as a highly promising approach, capable of selectively targeting specific cells by combining cell-targeted small interfering RNA (siRNA) with organ-specific biocompatible nanoparticles [66]. Research indicates that in mice with myocardial infarction, administering subcutaneous injections of liposome nanoparticles carrying myocardial infarction antigens and rapamycin leads to a reduction in cardiac inflammation and suppresses detrimental cardiac remodeling by fostering M2-like macrophage polarization, ultimately enhancing heart function [67].

Li *et al*. [68] developed a mesoporous silica nanoparticle system to deliver microRNA-21-5p, which effectively inhibits the polarization of M1 macrophages in infarcted myocardium, significantly reducing inflammatory responses and the area of myocardial infarction. Feng *et al*. [69] created a novel injectable hydrogel composed of puerarin and chitosan that uses *in situ* self-assembly to deliver mesoporous silica nanoparticles. This hydrogel suppresses M1 macrophage polarization, modulates the expression of pro-inflammatory cytokines, and facilitates myocardial repair. Xu *et al*. [70] devised a platelet membrane nanocarrier (PL720) that encapsulates L-arginine and FTY720 (FTY720 is an immunomodulatory drug approved by the Food and Drug Administration for clinical use in humans). This carrier specifically targets and releases these agents at sites of myocardial injury, effectively inhibiting cardiomyocyte apoptosis, enhancing myocardial survival, reducing fibrosis, and improving cardiac contraction and relaxation functions.

Recent research indicates that exosome-delivered microRNA therapies and nanomaterial drug delivery methods are promising as future treatments for targeting macrophage modulation following MIR, managing inflammatory responses, and ameliorating myocardial fibrosis. These approaches also bring new hope for the treatment of MIR injury, aiming to maximize the improvement of clinical outcomes for patients suffering from myocardial infarction.

## 7. Conclusions

Recent studies have highlighted the pivotal role of macrophages in injury response, tissue repair, and remodeling following MIR. Cardiac macrophages and their molecular mechanisms have increasingly been recognized as critical therapeutic targets for mitigating myocardial injury and preventing adverse remodeling after MIR. Post-MIR, these macrophages are essential for clearing cellular debris and facilitating tissue repair. However, excessive activation and infiltration of macrophages can exacerbate myocardial injury and contribute to pathological remodeling. The activities of various macrophage phenotypes during MIR can be contradictory, sometimes opposing each other, while at other times, they may act synergistically. A key focus in myocardial remodeling post-MIR is understanding the roles of different macrophage phenotypes throughout the stages of myocardial injury, particularly the interactions between different macrophage phenotypes with myocardial fibrosis, to develop effective therapeutic strategies against post-MIR myocardial fibrosis.

Recent investigations suggest that enhancing M2 macrophage polarization can significantly improve cardiac tissue repair in mouse models of MIR, with embryonic-origin CRMs showing substantial promise in aiding myocardial recovery [71]. Although we have made significant progress, there are still many areas that require further in-depth research, such as the more precise modulation methods in the transformation and regulation processes of different complex phenotypes of macrophages after MIR, which mechanisms dominate in the role of macrophages post-MIR injury, and how to more accurately deliver drugs to cardiac macrophages while minimizing MIR injury. Furthermore, it is equally crucial to determine when and where to minimize MIR injury in future clinical work, as well as to validate the safety and efficacy of potential treatment strategies in humans. In summary, future efforts should further explore the various functions and regulatory mechanisms of different cardiac macrophage phenotypes, continually investigate the modulation and intervention of macrophages, identify new therapeutic approaches for myocardial fibrosis post-MIR, and bring new hope for personalized medicine in treating cardiovascular diseases, thereby making a greater contribution to protecting cardiac health.

## Abbreviations

MIR, myocardial ischemia-reperfusion; PCI, percutaneous coronary intervention; MI, myocardial infarction; CRMs, cardiac resident macrophages; IMs, infiltrative monocyte-derived macrophages; ECM, extracellular matrix; Sectm1a, secreted and transmembrane protein 1a; DTR, diphtheria toxin receptor; Lgmn, legumain; GMCSF, granulocyte-macrophage colony-stimulating factor; MSCs, mesenchymal stem cells; MSC-exos, MSC-derived exosomes; TLR4, toll-like receptor 4.
